# Cellular and Molecular Mechanisms of Mast Cells in Atherosclerotic Plaque Progression and Destabilization

**DOI:** 10.1007/s12016-024-08981-9

**Published:** 2024-01-30

**Authors:** Daniel Elieh-Ali-Komi, Ilze Bot, Mónica Rodríguez-González, Marcus Maurer

**Affiliations:** 1 Institute of Allergology, Charité–Universitätsmedizin Berlin, Corporate Member of Freie Universität Berlin and Humboldt-Universität zu Berlin, Berlin, Germany; 2 Fraunhofer Institute for Translational Medicine and Pharmacology ITMP, Immunology and Allergology, Berlin, Germany; 3 Division of BioTherapeutics, Leiden Academic Centre for Drug Research, Leiden University, Leiden, The Netherlands; 4 Allergology Department, Hospital Español, Mexico City, México

**Keywords:** Cardiovascular disease, Mast cells, Atherosclerosis, Inflammation, Plaque rupture, Degranulation

## Abstract

Mast cells (MCs) are commonly recognized for their crucial involvement in the pathogenesis of allergic diseases, but over time, it has come to light that they also play a role in the pathophysiology of non-allergic disorders including atherosclerosis. The involvement of MCs in the pathology of atherosclerosis is supported by their accumulation in atherosclerotic plaques upon their progression and the association of intraplaque MC numbers with acute cardiovascular events. MCs that accumulate within the atherosclerotic plaque release a cocktail of mediators through which they contribute to neovascularization, plaque progression, instability, erosion, rupture, and thrombosis. At a molecular level, MC-released proteases, especially cathepsin G, degrade low-density lipoproteins (LDL) and mediate LDL fusion and binding of LDL to proteoglycans (PGs). Through a complicated network of chemokines including CXCL1, MCs promote the recruitment of among others CXCR2^+^ neutrophils, therefore, aggravating the inflammation of the plaque environment. Additionally, MCs produce extracellular traps which worsen inflammation and contribute to atherothrombosis. Altogether, evidence suggests that MCs actively, via several underlying mechanisms, contribute to atherosclerotic plaque destabilization and acute cardiovascular syndromes, thus, making the study of interventions to modulate MC activation an interesting target for cardiovascular medicine.

## Introduction

### Atherosclerosis Pathology and Microenvironment of Plaques

Atherosclerosis is an inflammatory lipid-driven pathology underlying many cardiovascular and metabolic diseases. Its complex pathology includes lesion initiation, progression, rupture or erosion, healing, and consolidation [1]. Plaques are dynamic entities in terms of cytoarchitecture and molecular interactions. The presence of lipid metabolites and events such as foam cell formation, accumulation of immune cells, and the release of proinflammatory mediators establish an inflammatory environment in the plaques [2]. Rupture of a plaque may lead to severe consequences of which stroke and myocardial infarction have a high medical significance [3]. The microenvironment of plaques is populated by a variety of cell types including endothelial cells, smooth muscle cells (SMCs), macrophages, lymphocytes, and MCs. These cells produce and release chemokines; cytokines; proinflammatory mediators such as IFN-γ, IL6, and TNF [4]; and proteases that may contribute to atherosclerotic lesion development [5, 6].

**Fig. 1:**
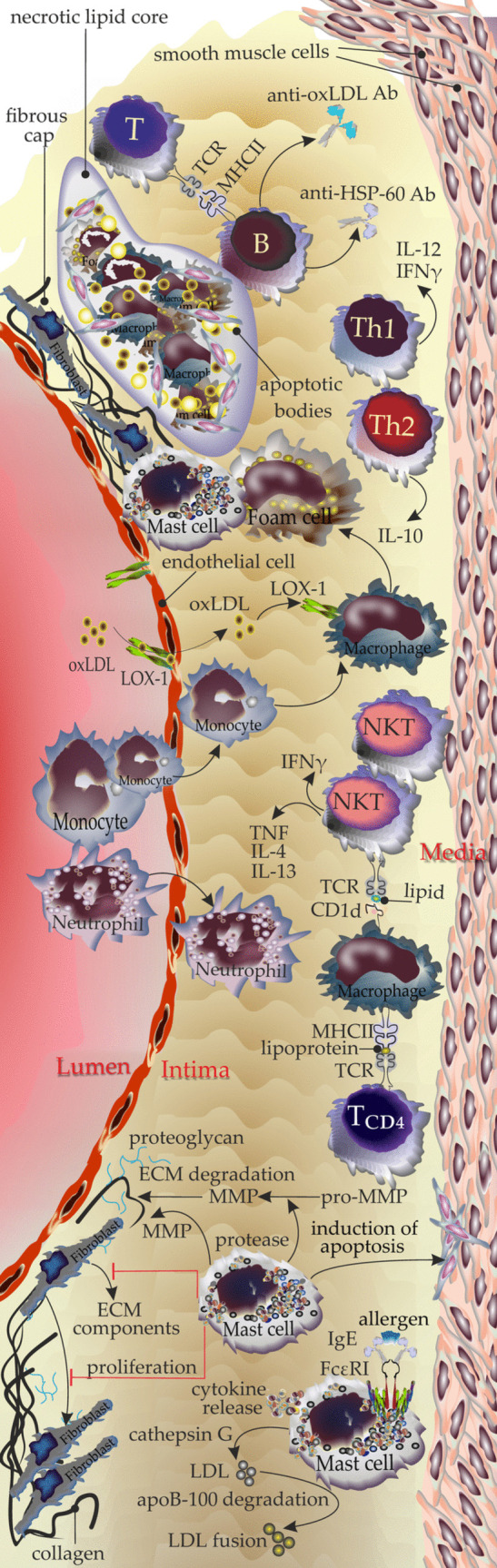
Microenvironment of atherosclerotic plaques and interactions between residing immune cells in brief. B cells become activated and produce antibodies including anti-oxLDL and anti-HSP-60 Abs. Th1 and Th2 cells produce IL-12, IFN-γ, and IL-4 respectively. Oxidized LDL is taken up through LOX-1 receptors expressed on endothelium and then is accumulated inside the macrophages to form foam cells. Th2 cells play an important role as atheroprotective immune cells. Th2-produced IL-4 counteracts the production of IFN-γ which is a proatherogenic cytokine; therefore, differentiation in favor of Th2 leads to modulating the production of atherogenic cytokines. NKTs recognize lipids presented by CD1d-expressing APCs that become activated and release IFN-γ, TNFα, IL-4, and IL-13. Macrophages act as APCs and present lipoproteins to CD4^+^ T cells. MCs accumulate within plaques during atherosclerosis and become activated and release proteases including cathepsin G that targets LDL. Tryptase and chymase released by MCs play a role in instability and rupture of the plaques. They release cytokines and recruit further immune cells into the plaque microenvironment. Additionally, mediators released by MCs inhibit the production of ECM components by fibroblasts and the proliferation of fibroblasts and induce the apoptosis of smooth muscle cells. The proteases released by MCs convert pro-MMPs to active MMPs which contribute to ECM degradation. MCs also act as the source of MMP

Atherosclerosis is triggered by endothelial damage of arteries and the deposition of low-density lipoproteins (LDL) [7]. Additionally, monocytes that enter the plaque differentiate into macrophages, which later play a key role in the pathology of the disease [8]. Endothelial cells transfer oxLDL to the intima by expressing lectin-like oxidized low-density lipoprotein receptor-1 (LOX-1) and exposing macrophages to oxidized low-density lipoprotein (oxLDL) [9]. This process drives the formation of foam cells leading to the initiation of atherosclerotic plaque formation [3]. Foam cells contribute to establishing the plaques by ingesting exceeding levels of lipids [10]. Moreover, macrophages present the peptide and lipid fragments to the surrounding T cells via MHC /TCR or NKTs through CD-1d /TCR interactions respectively [10–12]. Th1 cells act as proatherogenic cells by releasing IL-12 and IFNγ. Tregs are also held to play an atheroprotective role, by releasing IL-10 [13, 14]. The consequence of NKT activation in response to the stimuli is the release of cytokines including atherogenic Th1 cytokines such as IFNγ and TNFα as well as the Th2 cytokines IL-4 and IL-13 [13]. B cells can be, depending on their subset, either proatherogenic or atheroprotective. Their depletion may affect splenocyte proliferation in response to oxidized LDL suggesting a role for B cells in the presentation of lipid antigens to T cells [15]. Several autoantibodies such as anti-heat shock protein 60 (HSP-60) and anti-oxLDL have been reported to contribute to the pathology of atherosclerosis [15] (Fig. 1).

The histo-architecture of atherosclerotic plaques shows that they contain accumulations of SMCs and densely organized collagens covering the area with necrotic fragments, inflammatory cells, cholesterol clefts, and deposition of calcium [16]. Accumulation of immune cells and secretion of cytokines in favor of inflammation are of the key factors bridging the above-mentioned features. In this regard, macrophages accumulate and release inflammatory cytokines mainly TNF, IL-1β, and IL-6; induce the necrosis of cells within plaques; and produce matrix metalloproteinases in particular MMP-9 which degrade the extracellular matrix (ECM) and weaken the plaque stability [17, 18]. The stability of plaque can be affected by features especially the presence of large necrotic lipid-rich core, a thin fibrous cap, extension of vasculature, and hemorrhage that together make the plaque prone to rupture [17, 18]. The occurrence of atherosclerotic plaque rupture may result in arterial thrombosis, myocardial infarction, and stroke [16].

Considering our aim to highlight the role of MCs in the pathology of atherosclerosis, in the next paragraph, we review and highlight specific aspects of the disease and the main mechanisms of MC involvement in reshaping the atherosclerotic plaque microenvironment.

### MCs in Plaque Microenvironment

Investigation of the role of MCs in atherosclerosis goes back to the early 1950s, when Constantinides, based on previous studies showing heparin’s capability to remove lipoproteins from the blood of individuals with atherosclerosis and prevent lipidemia and that MCs are of the sources of heparin, focused on these cells to reveal the possible new aspect of pathophysiology of atherosclerosis [19].

From a histologic point of view, MCs are found in both the intima and perivascular tissue of atherosclerotic plaques [20]. Very early studies showed the presence of MCs in coronary arteries and provided information regarding their subtypes using monoclonal Abs against tryptase and chymase [6].

Kaartinen et al. showed that in the normal coronary intimas, 0.1% of all nucleated cells were MCs. The fatty streaks showed a higher MC density (ninefold), and in the cap, core, and shoulder regions of atheromas, MC density reached 5-, 5-, and tenfold, respectively when compared to normal coronary intimas. Using specific anti-tryptase Abs, they detected an average of 1 MC/mm^2^ in the normal intima, while a higher number of MCs (5 MC/mm^2^) was detected in intimas from individuals with atherosclerosis. Their results showed that the fatty streaks of normal intimas and the shoulder of atheromas contain a population of MCs of which an average of 35–40% was chymase^+^, whereas cap and core regions were populated by an average of 53% and 45%, respectively [6].

Considering that SMCs produce ECM components, MC-released chymase may induce the apoptosis of SMCs and also production and activation of TGF-β in these cells which in turn inhibits their proliferation through which may play a role in predisposing the plaque to weakening and later to rupture [21].

Moreover, microscopic observation showed that the number of activated MCs with signs of degranulation was significantly higher in the intimal areas (92% and 85% in the core and shoulder regions of atheromas respectively) which were degranulated, this being a sign of their activation, featuring the progression of atherosclerosis including fatty streaks, cap, core, and shoulder compared to normal intimal areas [6].

Investigation of MC presence in specimens collected from postmortem coronary arteries showed that MCs were accumulated at immediate site of rupture and formed 6% of nucleated cells, while their density was lower at adjacent atheromatous area (1%) or at intact intimal (0.1%).

Moreover, this study showed that a large percentage of MCs in each anatomical site was degranulated. Degranulated MCs were, for example, found at the site of erosion or rupture (86%), while 63% in the adjacent atheromatous area. The non-ruptured intima had the lowest percentage of degranulated MCs (27%) [22].

In another study, performed on 44 sections of aortas collected from autopsies using anti-tryptase and anti-chymase antibodies, the following results were obtained: (1) chymase^+^ MCs were more abundant in the non-elderly group, (2) a positive correlation between the number of chymase^+^ MCs and percentage of collagen (rS = 0.115, *P* = 0.006) and also between number of tryptase^+^ MCs and percentage of collagen (rS = 0.111, *P* = 0.008) was found at the site, and (3) a positive correlation between lipidosis and the number of tryptase^+^ MCs (rS = 0.117; *P* = 0.004) was reported [23].

After accumulation in the adventitial tissue and plaques [24], MCs act as inflammatory cells by releasing cocktails of proinflammatory cytokines [5]. Investigation of MC density (MCD) in human atherosclerotic plaques revealed that MCD was higher in unstable plaques than in stable plaques [25]. Mekke et al. studied human carotid atherosclerotic plaques and found that the highest MC numbers per square millimeter were observed in the symptomatic culprit lesion and the lowest in the symptomatic more distant segment, demonstrating a positive correlation between MC accumulation, symptomatic plaque segment, neovascularization, and subsequent inflammatory cell recruitment [26]. Interestingly, postmortem studies of individuals with coronary artery atherosclerosis showed that immunoglobulin E (IgE) and tryptase levels were elevated when compared to control deceased individuals who died because of non-cardiac reasons [5]. This is in line with studies performed by Willems et al., in which circulating tryptase levels were of predictive value for future cardiovascular events. Investigation of MC number in human carotid atherosclerotic lesions revealed high numbers, which correlated with plaque microvessel density and with future cardiovascular events [27].

Postinflammation plaque calcification is commonly reported in end-stage atherosclerosis. Osteogenic differentiation of arterial SMC is linked to vascular calcification. Since MCs interact with SMCs, their effects on phenotype reprogramming of SMCs have recently been investigated by Skenteris et al. They reported the colocalization of tryptase^+^ MCs and α-SMA/SOX9^+^ SMCs or α-SMA/ RUNX2^+^ SMCs. Next, they investigated the MC activation capacity of plaque medium on bone marrow–derived MC culture and found that, unlike supernatants of calcified plaques, the supernatant collected from highly calcified plaques could not induce MC activation and reduced the MC releasing of MIP-1α(CCL3) suggesting that calcification inhibits MC activation, while both resting or activated MCs induce SMC calcification [28].

## Interaction of MCs and Immune Cells in Atherosclerotic Plaques

### MC Interaction with T Cells

#### CD4^+^ T Cells

Early investigations of the atherosclerotic plaque microenvironment revealed the presence of activated T cells and antigen presenting cells (APCs) including macrophages that strongly express HLA class II molecules [29]. These plaque CD4^+^ T cells become activated and proliferate once exposed to for example oxLDL (a potential autoantigen found in atherosclerotic plaques) and produce IFN-γ and IL-4 [29]. IFN-γ activates macrophages, thus supporting inflammation, while IL-4 acts as a driver of B cell differentiation and antibody production [29, 30].

**Fig. 2:**
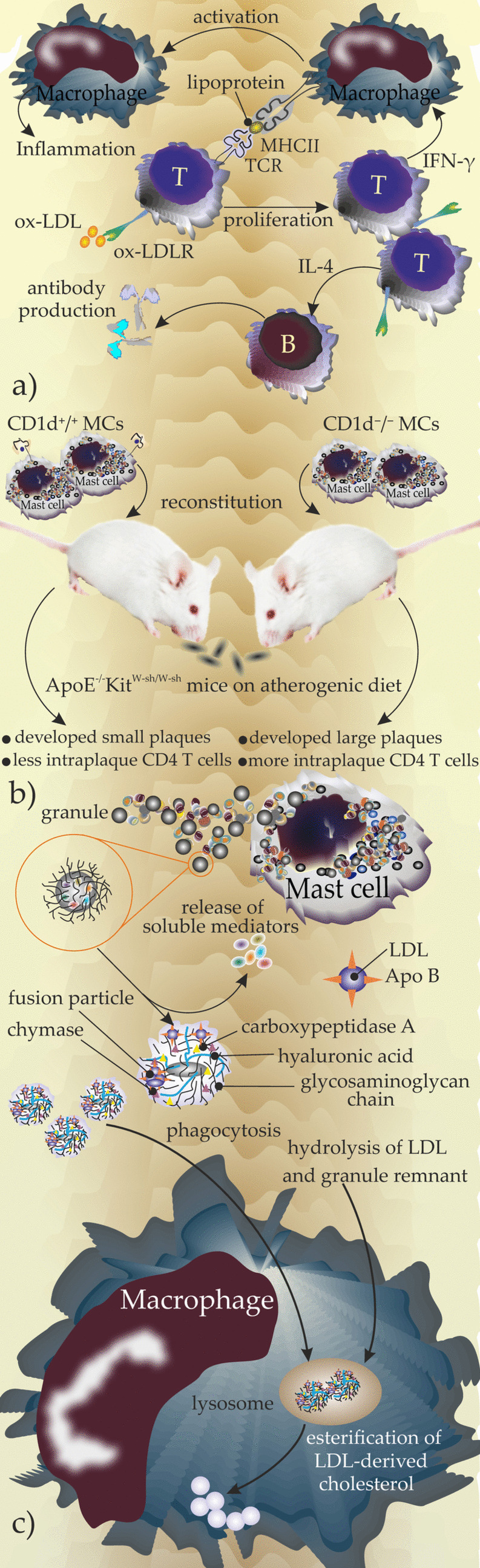
** a** Molecular mechanism of activation, releasing profile, and crosstalk of immune cells upon being exposed to oxLDL. CD4 + T cells become activated, proliferate, and release IFN-γ and IL-4 upon exposure to oxLDL. IFN-γ drives the activation of macrophages, therefore, supporting the inflammation, whereas, IL-4 induces B cell differentiation and antibody production. **b** Investigation of the role of CD1d − expressing or knockout MCs in mice on the atherogenic diet, reconstitution of apoE −/− Kit W−sh/W−sh mice with either CD1d −/− or wild-type CD1d +/+ MCs after being on an atherogenic diet showed that apoE −/− Kit W−sh/W−sh mice receiving CD1d −/− MCs develop larger plaques and have more intraplaque CD4 T cells. **c** An illustrated model of “granule-mediated uptake of LDL,” an interaction of MC-macrophage is based on exocytosis of granules from MCs which lose their soluble mediators and the remnants provide a scaffold to binding LDL by which ApoB will be detached from LDL and the remaining molecule fuse with other similar ones and eventually will be phagocytosed by macrophages. After trapping into lysosomes, LDL will be hydrolyzed and a final process of esterification of LDL-derived cholesterol occurs in the cytoplasm and the final lipid cargo is stored in macrophage and transformed into foam cells

Upregulation of MHCII molecules in MCs has been documented in the presence of IL-4 and IFN-γ (Fig. 2a). *Ldlr*^*−/−*^ mice with western-type diet (WTD) were reported to have increased levels of cholesterol, peritoneal MC (PMC) numbers, and upregulation of functional MHCII molecules as compared to mice on a low-cholesterol diet.

Besides, coculturing OT-II CD4^+^ T cells with WTD MCs loaded with OVA could induce the activation and proliferation of CD4^+^ T cells [7]. Investigation of the interaction of costimulatory molecules OX40L/OX40 expressed on MCs and T cells, respectively, in atherosclerosis provided another line of evidence for the role of MCs in the pathology of the disease. Treatment of mice with anti-OX40L (RM134L) Abs decreased the Th2 response, the levels of circulating IL-4 and IgE, and MC numbers in the plaque. *Ldlr*^*−/−*^mice receiving WTD treated with anti-OX40L Ab showed regression of atherosclerosis. This was linked to the production of IL-33 by APCs, which mediated the production of the atheroprotective cytokine IL-5 and oxLDL-specific IgM by T and B1 cells, respectively [31].

#### NKTs

MCs can present lipid antigens through their surface-expressed MHC class I–related molecule CD1d to CD1d-restricted natural killer T cells (NKTs), which activates them and induces the release of an array of cytokines [10, 32]. A recent study explored the role of the MC-CD1d/TCR-NKT axis in the pathology of atherosclerosis [10]. Kritikou and colleagues reconstituted MC-deficient apoE^−/−^Kit^W−sh/W−sh^ mice with either CD1d^−/−^ or wild-type CD1d ^+/+^ MCs and put both groups on an atherogenic diet. Their results showed that (1) apoE^−/−^Kit^W−sh/W−sh^ mice reconstituted with CD1d^−/−^MCs developed larger plaques when compared with apoE^−/−^Kit^W−sh/W−sh^ mice being reconstituted with WT MCs and (2) the density of intraplaque CD4^+^ T cells was higher in CD1d^−/−^MCs reconstituted apoE^−/−^Kit^W−sh/W−sh^. They concluded that the disruption of the MC-CD1d/TCR-NKT axis aggravates the progression of atherosclerosis [10] (Fig. 2b).

#### Neutrophils, Monocytes, Macrophages, and Foam Cells

Plaque-residing MCs have been reported to have a role in recruitment of other inflammatory cells to the cite. MCs degranulate after being activated by ROS released by the extravasated neutrophils. MCs then induce the recruitment of neutrophils and T cells by releasing TNF. Moreover, MC-released MCP-1 induces the recruitment of monocytes which later may develop in macrophage or foam cells and aggravate atherosclerosis [33].

Studies of the cellular architecture of atherosclerotic plaques showed a correlation between (a) the number of MCs with the percentage of the plaque area populated with macrophages (*r* = 0.156, *P* = 0.011) and (b) the number of degranulating MCs and the percentage of plaque residing macrophages (*r* = 0.310, *P* = 0.002) [27]. MC activation may contribute to the formation of foam cells. In line with this the term “granule-mediated uptake of LDL” was already introduced to picture how IgE-dependent MC activation results in the release of mediators that boost the macrophage LDL uptake. Granule-mediated uptake of LDL is defined as a process in which MC granules placing a variety of MC mediators promote after being released bind to LDL and phagocytosed by macrophages. The uptake of mediator LDL by macrophages results in enhancing the extent of cholesteryl ester synthesis in these cells and thus accumulation of cholesteryl esters [34].

**Fig. 3:**
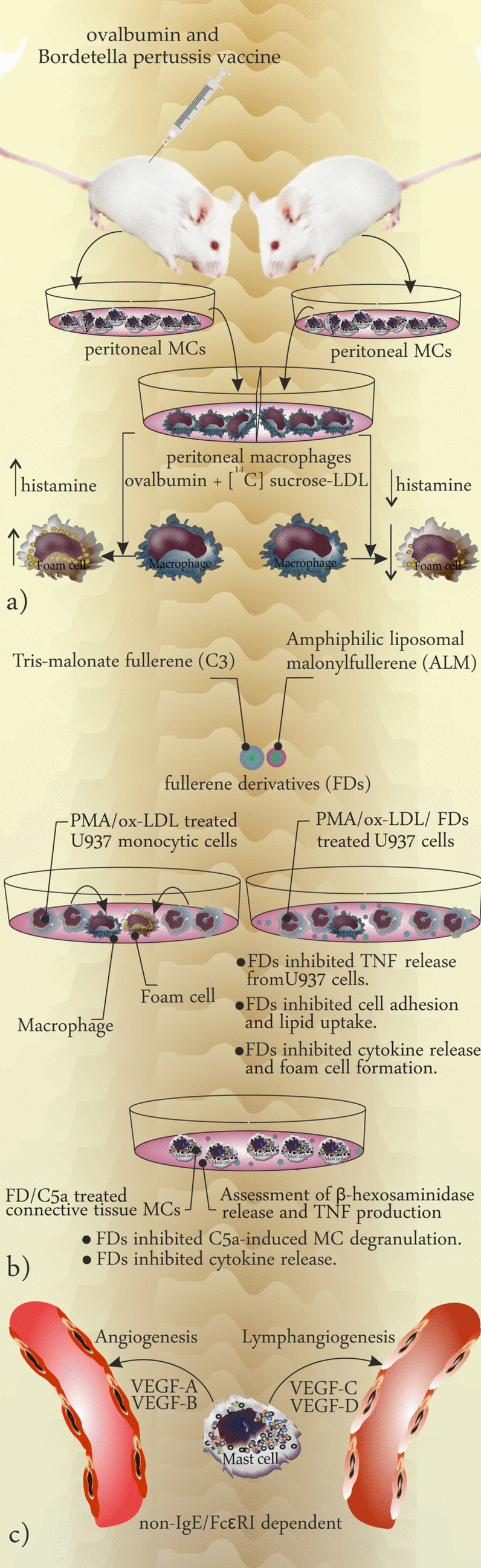
** a** Induction of foam cell formation using rat peritoneal MCs and macrophages. Peritoneal MCs and macrophages from immunized male Wistar rats with ovalbumin and Bordetella pertussis vaccine (as adjuvant) were harvested from treated and controls. Ovalbumin plus [ 14 C] sucrose-LDL were added to a monolayer culture of peritoneal macrophages and histamine levels in supernatant were measured. The results showed that the presence of ovalbumin-sensitized MCs boosts the capacity of macrophages to uptake LDL. In case MCs were not from immunized rats, the LDL uptake by macrophage was not significantly changed. Immunized MCs degranulated and released significantly higher levels of histamine and could induce the formation of foam cells more effectively. **b** Induction of foam cell formation from human myelomonocytic cell line in vitro: exposure of U937 cells to PMA followed by oxLDL resulted in the formation of foam cells. Addition of FDs inhibits the foam cell formation, TNFα release from U937 cells, cell adhesion, and lipid uptake. Β-Hexosaminidase and TNFα assessment in C5a-activated MCs and comparing the results with those obtained from C5a-activated MCs exposed to FDs revealed that FDs inhibit the degranulation and cytokine release. c Role of MC-released mediators in angiogenesis and lymphangiogenesis: MC-released VEGF-A and B induce angiogenesis, while VEGF-C and D induce lymphangiogenesis upon release in response to non-IgE/FcεRI-dependent activation

In this model described by Dr. Petri Kovanen in 1991, two cells are playing crosstalk: on the one hand, MCs degranulate IgE dependently and release granules that their soluble content releases upon exocytosis. Then, LDL attaches the glycosaminoglycan chains in remnant structure afterward, hydrophilic segments of apolipoprotein B are detached under influence of chymase and carboxypeptidase, and then proteolyzed LDLs fuse and phagocytosed by macrophage. Phagolysosomes of macrophages hydrolyze the complex and finally, esterification of LDL-derived cholesterol is completed in the cytoplasm [35, 36] (Fig. 2c). In a study performed by Ma and Kovanen, male Wistar rats were immunized with ovalbumin and *Bordetella pertussis* vaccine (as adjuvant) to induce the production of anti-ovalbumin IgE, after which peritoneal MCs and macrophages were harvested from ovalbumin-treated rats and controls to analyze macrophage LDL uptake and formation of foam cells. They prepared a monolayer culture of peritoneal macrophages, added ovalbumin-sensitized and non-sensitized-MCs, and then added ovalbumin plus [^14^C] sucrose-LDL to track the process of LDL uptake. Moreover, they measured histamine in the supernatant. They found out that the presence of ovalbumin-sensitized MCs boosts the capacity of macrophages to take up LDL. However, macrophage LDL uptake was not significantly changed in non-immunized conditions. They concluded that the interaction of MCs and macrophages to form foam cells is IgE dependent which results in degranulation of MCs. Liberation of histamine from the macrophage-phagocytosed granules induces the macrophage LDL uptake and mediates their transformation into foam cells [37] (Fig. 3a). On the other hand, activated MCs have been shown to induce macrophage apoptosis and plaque instability in advanced mouse atherosclerotic plaques, which was suggested to be mediated by MC proteases and histamine [38]. Both MC-macrophage interactions suggest that stabilizing MCs may provide a therapeutic approach to control atherosclerosis.

Apart from the in vivo models to study crosstalk between MCs and macrophage foam cells, in vitro studies have been described to study their potential interactions.

Plotkin et al. considering the anti-inflammatory effects of fullerene derivatives (FDs) investigated the effects of these water-soluble carbon spheres both on MC activation and foam cell (CD36^+^) formation from monocyte-derived macrophages (CD68^+^). The authors reported that FDs may act through the NF-κB pathway to render biologic effects including preventing the lipoprotein-induced release of TNF-α, upregulation of foam cell marker CD36, and C5a-mediated activation of MCs [3]. The expression of CD36, CD68, and scavenger receptor class A (SRA) enables macrophages to recognize dying cells and different forms of LDL mainly oxLDL [39]. Upon exposure to TNF-α, monocytes become activated and show signs of clumping and lipid uptake to form foam cells. In their experiment, PMA-treated cells clumped and showed a significant reactivity to Oil-Red-O staining. Application of FDs reverted these features in treated U937 cells [3]. Assessment of β-hexosaminidase release and TNF production in C5a-activated connective tissue MCs before and after FD treatment showed that FDs inhibited C5a-induced degranulation and cytokine production in MCs [3] (Fig. 3b).

## The Role of MCs in Neoangiogenesis and Vascular Remodeling in Atherosclerosis

MCs contribute actively to the processes of angiogenesis and lymphangiogenesis by releasing proangiogenic VEGF-A and VEGF-B, and prolymphangiogenic VEGF-C and VEGF-D, respectively [40, 41] (Fig. 3c). The production and release of VEGF are not IgE/FcεRI dependent (classic pathway of degranulation [42]) necessarily and MC-released IL-6 may induce the production of VEGF in an autocrine manner without inducing MC degranulation [43]. The role of MCs in the regulation of angiogenesis within atherosclerotic plaques was in the focus of Kaartinen and colleagues. They stained human coronary intima samples using Elastica-van Gieson to assess the formation of the plaques and applied anti-von Willebrand factor to screen the process of angiogenesis. Additionally, they benefited from monoclonal antibodies targeting MC tryptase and chymase to study MCs [44]. They reported the accumulation of MCs in areas with neoangiogenesis in the proximity of microvessels. Released tryptase and chymase from activated MCs and their angiogenic role are linked to the pathology of atherosclerosis [44]. Considering that coronary intima lacks the common capillary blood circulation, the invasion of microvessels to the deep regions of the atheromas supplies the plaques with necessary blood components and supports their progression [44]. Similarly, a significant association between mast cell numbers and microvessel density was shown in human carotid arteries, in which mast cells were shown in close proximity to intraplaque neovessels. Indeed, one could speculate that mast cells, upon activation, promote intraplaque hemorrhage due to the induction of microvessel leakiness [27]. Mast cells can also promote neovascularization upon the induction of ischemia, as was shown in a mouse model of hind limb ischemia [45].

Chillo and colleagues proposed a novel mechanism through which MCs may participate in arteriogenesis.

They considered that fluid shear stress, which drives the force behind arteriogenesis, is only directly detectable by vascular endothelial cells, but not by perivascular cells. They focused on platelets which sense the fluid shear stress and can adhere to endothelium-expressed von Willebrand factor (vWF) upon fluid shear stress increasing through platelet glycoprotein Ib (GPIb) receptor [33]. In their mouse model, blockage of the platelet receptor GPIba as well as genetic ablation of the ectodomain of GPIba in transgenic IL4-R/Iba mice inhibited MC degranulation to a level comparable to cromolyn treatment suggesting a functional link between platelet receptor GPIba and MC activation. Moreover, they found that in addition to platelet receptor GPIba, urokinase plasminogen activator (uPA), a serine protease that plays important roles in cancer invasion and cell migration [46] mediated extravasation of neutrophils [33]. Neutrophils after extravasation were shown to produce reactive oxygen species (ROS) by neutrophil-expressed Nox2 and then release these active molecules which in turn activate MCs [33]. They applied different techniques to deplete neutrophils (using Ly-6G (1A8) antibody), deficiency of Nox2, and blocking ROS production to investigate the effect of neutrophils and their produced ROS in activation of MCs and concluded that neutrophil-produced ROS mediate MC activation in their mouse model. Additionally, MCs activate matrix metalloproteinases (MMPs, zinc-containing endopeptidases involved in ECM turnover [47, 48]) by releasing proteases and play a role in vascular remodeling upon arteriogenesis [33].

## Role of MCs in Atherosclerosis Plaque Calcification

Intimal calcification is a clinical marker of atherosclerosis and although it has been recognized as an active vascular inflammatory response and remodeling, its role remains unclear in terms of plaque stability and prognosis due to differences in size and morphology [49]. Previous studies provided a line of evidence on MC involvement in the process. As an example, a study on 250 samples of atherosclerotic lesions in carotid arteries using anti-tryptase IHC investigated the association of MCs, macrophages, SMCs, and elastin with the extent of calcification. The researchers found that MCs and their released tryptase were largely associated with areas of early calcification (including stippling and morula-type calcifications) although MC accumulation and release of tryptase were observed in some late-stage solid calcifications. Additionally, the tryptase was associated with local matrix disruption and oedema at the matrix–calcification interface [50].

The study by Skenteris et al. demonstrated that unstable carotid atherosclerotic plaques and vascular lesions are abundant with activated MCs and that the average number of MCs was correlated negatively with the calcification content [51].

## Engaged MC Expressed Receptors in Atherosclerosis

### FcεRI

IgE/FcεRI accounts for the main mechanism of MC activation, where FcεRI molecules are engaged with allergen-bound IgE [52, 53]. This crosslink triggers the activation of protein tyrosine kinase Syk which leads to downstream events including the phosphorylation of several targets including TRAPs and LAT. “Phospholipase Cγ” (PLCγ) after anchoring and becoming activated catalyzes the “phosphatidylinositol 4,5-biphosphate” (PIP2) hydrolysis to form second messengers “diacylglycerol” (DAG) and inositol 1,4,5, -triphosphate (IP3). IP3 binds to its receptors on the endoplasmic reticulum (ER) and promotes Ca^2+^ efflux from the ER that results in MC degranulation [54]. IgE-FcεRI network triggers the release of MC mediators including histamine, tryptase, and chymase which play a role in the progression of plaques and are discussed in the following section. In human atherosclerotic plaques, MCs have been shown to express FcεRI, and a subset of these MCs had IgE molecules bound in their surface, suggesting that this may be an important activation pathway within the plaque. The local antigen(s) however, have not been identified [63].

According to the literature, there are studies that associate above-normal IgE levels with endothelial dysfunction and CVD in humans and animals [55]. In line with this, atopic patients with no sign of atherosclerosis with higher IgE levels had notably lower coronary flow reserve (CFR) which is used as an endothelial dysfunction biomarker [56]. In another study, higher levels of serum IgE levels were found in patients with acute ischemic stroke [57]. Moreover, an association between higher IgE levels with cardiovascular mortality was reported [58].

Interestingly, Wilson et al., after evaluating the total and specific IgE to a food allergen (α-Galactose) in 118 subjects, found a correlation between total IgE and α-Gal specific IgE levels and between α-Gal-specific IgE levels and the atheroma burden [59].

Another aspect of IgE in exacerbation of atherosclerosis was reported when natural secreted IgM (sIgM) was studied. In the context of atherosclerosis, these Abs target oxidation-specific epitopes found on OxLDL; therefore, functionally anti-OxLDLsIgM was suggested to be protective in atherosclerosis and CVD mainly by neutralizing proatherogenic effects of OxLDL. Investigation of B cell generation in sIgM^−/−^and Ldlr^−/−^sIgM^−/−^ revealed that these cells have an impaired generation; therefore, the IgE clearance mediated by their expressed low-affinity IgE receptor (CD23) was not performed normally resulted in an increase in IgE levels. Application of IgE-neutralizing Ab (R1E4) reduced vascular inflammation and limited atherosclerotic lesion formation. Among the results reported in this study, MCs in Ldlr^−/−^sIgM^−/−^ mice had a higher rate of activation visualized by chloroacetate esterase staining when compared to Ldlr^−/−^ mice probably due to higher levels of IgE [60].

Bruton’s tyrosine kinase (BTK) is expressed in B cells and MCs. In atherosclerosis, follicular B cells are described to exert atherogenic functions, such as proinflammatory cytokine production, IgG antibody secretion, and regulating T cell responses. In MCs, upon IgE to FcεRI binding, downstream proteins Lyn and Syk become phosphorylated, resulting in the phosphorylation of BTK. Phosphorylated BTK leads to phosphorylation of PLCγ2 and eventually leads to mast cell degranulation and production of inflammatory cytokines.

Considering the crucial role of FcεRI signaling in the responsiveness of MCs to IgE, Hemme et al. applied a Bruton tyrosine kinase inhibitor (acalabrutinib) to study the significance of the FcεRI receptor signaling in progression of atherosclerosis in Ldlr^− /−^ mice on high-fat diet (HFD) 2 months while taking acalabrutinib. They observed an impaired B cell maturation (as BTK is a necessary factor in the maturation of B cells [61]), a significant increase in splenic immature follicular II B cells in acalabrutinib-treated mice, and a decrease in mature follicular I B cells, whereas MC numbers and activation remained unaffected, and the size of plaques was not altered [62].

These data confirm that acalabrutinib successfully inhibited BTK in vivo and that BTK inhibition leads to a shift towards less B cell maturation in atherosclerosis. Despite the effect on B cell maturation, circulating antibody levels remained unaffected. They concluded that BTK inhibition alone did not affect MC activation in early and advanced stage atherosclerosis, despite the systemic biological effect on follicular B cell maturation [62].

IgE/FcεRI-mediated activation of MCs in plaques has been also reported and rationalizes a molecular mechanism involved in the pathology of Kounis syndrome [63] in which cardiovascular symptoms occur secondary to allergic or hypersensitivity insults. In this unrecognized or undiagnosed cardiovascular pathology due to its broad clinical manifestations, the symptoms are initiated by a number of reasons mainly drugs, environmental exposures, nutrients, and coronary stents. Pathologic events such as coronary spasm, acute myocardial infarction, and stent thrombosis, accompanied by activation of MCs and platelets and the presence of inflammatory cells are the main findings of Kounis syndrome [64, 65].

### Complement Receptor

The activation of the complement system has been documented in atherosclerosis, and high levels of C5a have been reported in individuals suffering from the disease. MCs become activated and degranulate upon C5a/CD88 binding [3]. In an in vivo experiment, C5a-mediated MC activation was shown to enhance atherosclerotic vein graft disease in a MC-dependent fashion [66].

### TLRs

MCs express both endosomal and membrane toll-like receptors (TLRs) [67] of which TLR-4 responds to pathogens by sensing their LPS. It is now evident that LPS activation of MCs induces the release of chymase and IL-6 that destabilize plaques and induce the apoptosis of vascular SMCs in plaques, respectively [68].

### Neuropeptide Receptors

Among the activating receptors expressed on MCs, MRGPRX2 makes MCs responsive to neuropeptides including substance P (SP) [69]. However, the expression of MRGPRX2 awaits to be explored on cardiac MCs, and responsiveness of MCs could be due to the engagement of other neuropeptide receptors. Neurokinin-1 receptor (NK1R) acts as a receptor for SP and is expressed on MCs. The presence of plaque-residing MCs in close vicinity of nerve fibers capable of releasing SP inspired Bot et al. to inspect the SP-mediated MC activation in a SP-treated WTD fed apoE^−/−^ mice after 6 weeks of placing semiconstrictive collars at the carotid arteries. They reported that SP treatment increases adventitial MC numbers, activates MCs, and promotes intraplaque hemorrhage. Application of neurokinin-1 receptor antagonist spantide I could prevent the effects suggesting that SP induces MC activation [70].

### Lysophosphatidic Acid (LPA) Receptor

It is evident that lysophosphatidic acid (LPA), a MC activator, progressively piles up in plaques during the progression of atherosclerosis and in vitro investigation of its potency on degranulation of a variety of MC types including MC/9 cells, BMMCs, and mouse PMCs. In vitro investigation showed that LPA induces β-hexosaminidase and tryptase release from these cells. Additionally, intradermally injection of LPA or LPA-activated MCs could induce vascular leakage, which destabilizes plaque, while this effect and the release of tryptase were abolished when LPA was injected to Kit^W−sh/W−sh^ mice. Application of this in vitro model of LPA-mediated MC activation to a mouse in vivo model provided a link to study the effects of LPA accumulation over time on activation of plaque-residing MCs and then the study of LPA-MC axis in atherosclerosis. Bot and colleagues then applied LPA perivascularly using a gel at the collar-induced carotid artery lesion in apoE^−/−^ mice. Although no plaque size variation was observed between the LPA-challenged and control mice after 3 days, the number of activated MCs was notably higher (*P* < 0.05) in the LPA-challenged mice when cromolyn was applied [71].

## Involvement of MC-Released Mediators in Atherosclerosis

### Histamine

One mechanism by which cellular cholesterol homeostasis is maintained is reverse cholesterol transport (RCT). During this process, cellular cholesterol is transferred from peripheral organs to the liver to undergo fecal excretion as bile acid [72, 73]. MC-released histamine induces macrophage RCT in an H1R-mediated pathway. Via this receptor. histamine can disrupt the endothelial barrier and facilitate the transfer of atheroprotective HDL into interstitial fluid and then the liver [72].

### Protease

Activated MC_TC_s release tryptase and chymase and have been reported to accumulate at the site of rupture [38]. Moreover, MCs contribute to the aggravation of the inflammation and destabilization of coronary plaques by releasing TNF-α [38]. Cathepsin G possesses serine protease activity and is produced mainly by MCs and neutrophils [74]. It induces the elastin solubilizing activity of elastase and activation of several MMPs including MMP-1, MMP-2, MMP-3, and MMP-9 [74, 75]. Investigating the role of cathepsin G in the induction of atherosclerosis in *Ldlr*^*−/−*^ mice on WTD showed that cathepsin G acts as an atherogenic factor in the early stages of the disease; however, it degrades LDL (having no effect on HDL or triglyceride content) through which it plays a protective role in the progression of atherosclerosis [75]. Maaninka et al. studied the role of MC-released proteases in the pathology of atherosclerosis. They incubated LDL with MC-conditioned medium prepared by activating human MCs with calcium ionophore and studied the binding of modified LDL to isolated aortic proteoglycans or to atherosclerotic plaques ex vivo. Their results showed that of the released proteases including tryptase, chymase, carboxypeptidase A3 (CPA3), granzyme B (GrB), and cathepsin G, only the latter showed degradation of apoB-100, induction of LDL molecule fusion, and promoting the binding of LDL to aortic proteoglycans and lesions [76].

MC-released chymase is capable of proteolytically cleaving the carboxyl terminal of apolipoprotein A-I (apoA-I). This capability of chymase makes apoA-I unable to bind to coronary artery endothelial cells (ECs). Additionally, other outcomes of enzymatic effects of chymase on apoA-I include suppression of nuclear factor-κB (NF-κB)–dependent upregulation of vascular cell adhesion molecule-1 (VCAM)- and abrogation of apoA-I anti inflammatory activities such as downregulation of the expression of TNF-α, IL-1β, IL-6, and IL-8 in foam cells and inhibition of ROS formation in neutrophils [77]. A Langendorff heart perfusion system-based study showed that compound 48/80 activated rat cardiac MC release chymase that degrades apoA-I at Tyr^192^ and Phe^229^ sites. Interestingly, under hypoxia-mediated MC activation, chymase was reported to cleave 243-amino acid intact apoA-I only at Tyr^192^ site [78]. The Tyr^192^-truncated fragment in proportion to the intact apoA-I showed significantly lower ability to mediate the migration of cultured human coronary artery endothelial cells during in vitro wound-healing test [78].

SMCs play a crucial role in the production of matrix and their loss is a feature that predisposes the plaque to weakening and later to rupture. The results of in vitro studies showed that MC-produced chymase could inhibit the collagen type I and III mRNA expression SMCs in cultured rat and human coronary arterial SMCs. Moreover, chymase induces the production and activation of TGF-β in SMCs which in turn inhibits their proliferation and growth through arresting G0/G1 → S transition. Application of anti-TGF-β antibody could partially reverse the inhibitory effect [21]. MC-produced chymase can directly induce apoptosis in growth-arrested rat arterial SMCs treated for 4 h with chymase. The mechanism is based on the disruption of NF-κB signaling resulting in bcl-2 mRNA downregulation, influencing the expression of protein, and leakage of cytochrome c [79].

Mouse MC protease-4 (mMCP-4) is the mouse protease mostly related to human chymase and its involvement in the progression of atherosclerosis has been studied. The results of Houde et al. on ApoE^−/−^, mMCP-4^−/−^, and ApoE^−/−^mMCP-4^−/−^ mice in different stages of atherosclerosis showed that mMCP-4 inhibition may reduce plaque progression in the earlier phases of atherosclerosis and stabilize the advanced plaques [80].

### Cytokines and Chemokines

Additionally, MCs contain a complex array of chemokines that mediate the recruitment of immune cells, of which, for example, neutrophils aggravate the inflammatory status of the plaques. For instance, (MC-released) CXCL1/(neutrophil expressed) CXCR2 interaction provides a mechanism through which neutrophils may be recruited into the plaque environment [20]. Mouse MCs released chemokine KC (ortholog of human IL-8, also known as a cytokine-induced neutrophil chemoattractant (CINC), growth-related protein α (Gro-α), and CXCL1 [81]) acts through monocyte receptors CXCR2 and VLA-4 and plays a role in the recruitment of monocytes to the plaques [38].

**Fig. 4:**
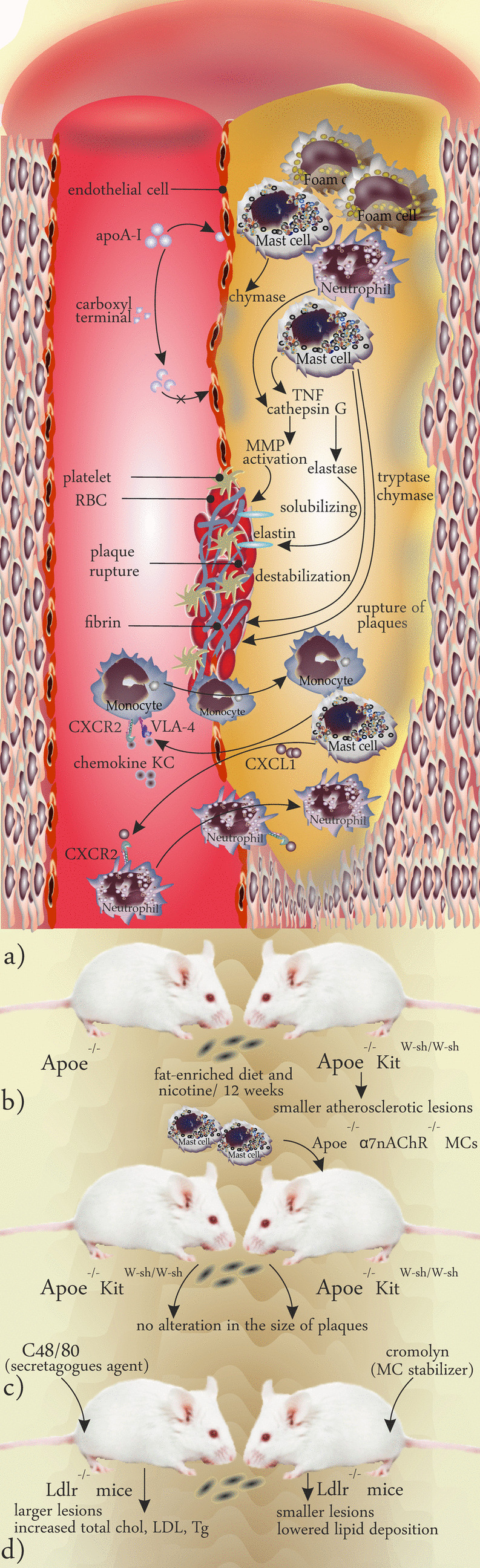
** a** Role AQ of MC-released mediators in plaque microenvironment. MC-released chymase degrades ApoA-I and prevents its binding to endothelial cells. Both neutrophils and MCs produce cathepsin G that activates elastase which in turn solubilizes the elastin and activates MMPs. Both tryptase and chymase destabilize the plaque and contribute to rupture. Additionally, MCs play a role in recruiting immune cells into plaques., i.e., chemokine KC (CXCL1) in mice acts through CXCR2 and VLA-4 and recruits the monocytes into plaques, and CXCL1 released from MCs acts through neutrophil expressed CXCR2 and recruits them. **b** Investigation of the plaque progression in Apoe −/− mice and Apoe −/− Kit W−sh/W−sh after a fat-enriched diet showed that Apoe −/− Kit W−sh/W−sh develop smaller lesions. **c** To explore whether α7 nicotinic acetylcholine receptor (and binding to nicotine) plays a role in the development of plaques, Apoe −/− Kit W−sh/W−sh mice were reconstituted with MCs isolated from Apoe −/− α7nAChR −/− mice and compared with Apoe −/− Kit W−sh/W−sh mice as controls after putting them on an atherogenic diet. No significant difference was found between the size of the plaques between the two groups showing that deficiency in α7 nicotinic acetylcholine receptor hampers the atherogenic effects of nicotine on the progression of plaques. **d** Comparing the effects of induction of MC activation and stabilization using C48/80 and cromolyn in an Ldlr −/− mouse model fed an atherogenic diet showed that MC activation results in larger lesions and increased levels of cholesterol, LDL and triglycerides, while application of MC stabilizer showed converse results

Results obtained from mouse models revealed that MC-derived IL-6 and IFNγ play a role in the progression of atherogenesis [82]. The ability of plaque-residing MCs in the production of proinflammatory cytokines was also reported in human studies too. Kaartinen et al., after studying the TNF positivity in atheromas obtained from 37 postmortem collected coronary arteries, concluded that MCs were the main cells producing TNF and were distributed differently in atheromas in which above half of the TNF-positive MCs were found in the shoulder region, 35% in the cap, and 10% were in the core regions. Visualization of TNF in MCs showed that this proinflammatory mediator is placed within the granules which enables MCs to release it upon degranulation [83] (Fig. 4a).

## Application of Mouse Models in Studying the MC Involvement in Atherogenesis: Lessons from Animal Models

Nicotine is an addicting component in cigarettes and has atherogenic properties. Wang et al. provided a MC-dependent link between smoking and atherosclerosis. Nicotine possesses atherogenic properties, and exposure to nicotine may increase the size of plaques and increases the levels of proinflammatory cytokines including IL-4, IL-6, TNF-α, and IFN-γ. They compared the progression of plaques between apolipoprotein E-deficient mice (Apoe^−/−^) and MC-deficient Apoe^−/−^Kit^W−sh/W−sh^ mice after they were fed a fat-enriched diet and received nicotine for 12 weeks and showed that the latter had decreased atherosclerotic lesion size (Fig. 4b). They concluded that MCs may aggravate atherosclerosis and that nicotine not only increases the density of MC density but also activates them. Additionally, their experiment on activation of bone marrow–derived MCs by nicotine in vitro showed that the α7 nicotinic acetylcholine receptor may be the receptor through which nicotine activates MCs. To test this mechanism, they reconstituted Apoe^−/−^Kit^W−sh/W−sh^ mice with MCs of Apoe^−/−^α7nAChR^−/−^ mice and reported no alteration in the size of plaques suggesting that MC activation by nicotine is achieved by engaging α7 nicotinic acetylcholine receptors [84] (Fig. 4c).

One important aspect of studying the role of MCs in the pathology of atherosclerosis could be monitoring the progression of the disease in the presence of MC stabilizers and secretagogues. Wang et al. monitored atherosclerosis in Ldlr^−/−^ mice fed an atherogenic diet who received either the MC secretagogue compound 48/80 (C48/80) or the MC stabilizer cromolyn. They found that the application of C48/80 could cause histologic alterations including increasing aortic arch intima and total lesion areas and biochemistry changes such as elevated levels of total cholesterol, LDL, and triglyceride. As expected, cromolyn acted conversely and lowered the lipid deposition of thoracic-abdominal aortas and prevented the activation of MCs [85] (Fig. 4d). Sun and colleagues studied the microenvironment of atheromata in Ldlr^–/–^Kit^W−sh/W−sh^ mice and reported decreased lesion size, reduction in lipid deposition, and low numbers of macrophages and T cells. To elucidate the role of MC-produced mediators in the pathology of atherosclerosis, they used an adoptive transfer strategy using MCs obtained from either syngeneic WT, IL-6^−/−^, IFNγ^−/−^, or TNFα^−/−^ mice to restore atherogenesis in Ldlr^–/–KitW−sh/W−sh^ mice. They concluded that MC-derived IL-6 and IFNγ actively participate in the progression of atherogenesis in mice [82].

Lagraauw and colleagues reported that acute stress may have a role in the activation of MCs. Using a stress protocol, they studied the outcomes in apoE^−/−^ mice and reported that (a) circulatory leukocytes shift in favor of neutrophils, (b) glucocorticoid levels increase, (c) perivascular MCs under stress become activated and degranulate, and (d) the levels of MC-derived mediator including β-hexosaminidase increase and correlate with the number of activated MCs [86].

## Unmet Questions in MC Involvement in Atherosclerosis

In this section, we summarize a list of unmet questions with the corresponding rationale for MC involvement in the pathology of atherosclerosis that need further investigations (Table 1).

**Table 1:** Summarizing the rationale corresponding to possible unmet questions for prospective themes of research

Unmet questions in MC involvement in atherosclerosis	Ref
** > > > Can MCs in atherosclerotic plaques change their phenotype? If so, what are the drivers and consequences?** Investigation of accumulated MCs in atheromas revealed that the majority of them are tryptase positive while expressing variable levels of chymase (0–100%). The outcome of the presence of either MC_T_ or MC_TC_ subtype may alter the involvement of MCs in the pathology of the disease	[6]
** > > > How are mast cell proteases involved in atherosclerosis and what is their value as biomarkers?** Measuring the serum levels of tryptase in acute coronary syndromes has been reported to be beneficial in predicting and following up cardiovascular events. According to the mechanism of MC involvement in chronic vascular diseases including atherosclerosis, measurement of tryptase as the specific MC-secreted enzyme may be helpful in the prediction of further events and progression of the disease. Additionally, the assessment of carboxypeptidase A3 in diseases with vessel damage showed that lower levels of the enzyme are associated with higher risk and damage	[87, 88]
** > > > What are the allergens/autoallergens targeted by the IgE that is bound to MCs in atherosclerotic plaques?** Defining the stimuli in atherosclerotic plaques that drive MC activation may help to develop novel treatments that target them. In this regard, Kritikou et al. using a novel flow cytometric method reported the presence of an activated MC population with CD63 expressing (a lysosomal marker that fuses the membrane upon MC activation and degranulation) within a plaque microenvironment with surface IgE-bounded FcεRI. IgE-dependent activation of MCs links to allergic disorders and autoallergy	[89]
** > > > How do comorbid diseases affect MC numbers and function in atherosclerosis?** The presence of other diseases should be considered in studying atherosclerosis due to their possible effect on plaque cellularity. In this regard, investigation of MCs and DCs in individuals with both atherosclerosis and chronic kidney disease (CKD) (immunosuppression and impaired T cell immune response are findings in individuals with CKD) showed a higher number of accumulated MCs but fewer DCs compared to the control number especially in calcified plaques suggesting a proinflammatory role for plaque resident MCs. The effects of other chronic disorders on the characterization of plaques in terms of cellularity and immune responses remain unclear	[90]
**> > > What is the relevance of MC interactions with DCs in the formation of atherosclerotic plaques?** Investigation of the cellular crosstalk between MCs and other plaque infiltrating cells with atherogenic activity like myeloid DCs may provide more details on how MCs involve the pathology of the disease	[91]
** > > > How does MC depletion or silencing affect the course of atherosclerosis?** Disrupting KIT signaling using tyrosine kinase inhibitors (TKIs) including imatinib affects the late stage of MC differentiation and depletes MCs. Several studies reported promising effects of imatinib in the treatment of atherosclerosis and abdominal aortic aneurysm. Moreover, imatinib-loaded nanoparticles have been formulated for further studies in the treatment of atherosclerosis. Studying the possible effects of other TKIs and therapeutic antibodies such as CDx-0159 may provide another pharmaceutical approach for MC targeting in atherosclerosis	[92–96]
** > > > Do MC-activating signals, in patients with atherosclerosis, come with an increased risk of MI? If so, how can this risk be reduced?** Because MCD is significantly increased in unstable lesions compared to stable lesions after MI, their possible involvement in MI needs further attention. In a rat model, it was shown that pretreatment with propofol, an anesthetic agent possessing antioxidant properties, C48/80-mediated MC activation, and exacerbation of myocardial ischemia–reperfusion can be suppressed	[97, 98]
** > > > Is there a role of MCs in the initiation of atherosclerosis in humans after major orthopedic surgeries?** Major surgery represents one of the non-infectious conditions that lead to systemic inflammation and has been shown to increase the risk of developing atherosclerosis in humans. For instance, it has been reported that the risk of acute MI increases after total knee replacement. The possible role of MCs in triggering these events is unclear Investigation of MCD showed that the MC population did not change in ApoE^−/−^ mice fed with an atherogenic diet and has undergone femur osteotomy although the development of plaques was significantly induced; however, measuring the serum amyloid A as a systemic inflammation marker showed a notable increase. The role of MCs in the increased rate of postsurgery cardiovascular events in humans, contribution to establishing an inflammation state, their activation, and serum levels of MC mediators await to be investigated	[99, 100]
** > > > What is the role of extracellular trap formation by MCs in atherosclerosis and what drives them?** MCs form extracellular traps in the plaque environment; however, the triggering stimuli remain to be clarified	[101, 102]
** > > > Is mastocytosis linked to atherosclerosis?** Mastocytosis is defined as a clonal infiltration of neoplastic MCs into internal organs or the skin. Significantly elevated levels of tryptase and chymase in these individuals are associated with plaque destabilization. Cardiac arrests and acute MI are often reported cardiac events in individuals with systemic mastocytosis (SM). Identifying the activating stimuli may help to have a better insight into the neoplastic MCs reaction and comparing the results to those of normal non-neoplastic perivascular residing MCs. In this regard, Indhirajanti et al. reported that although their studied individuals with SM had lower LDL and total cholesterol levels compared to the normal group, they had a higher rate of cardiovascular diseases	[103–107]
** > > > Does crosstalk between immune cells in terms of establishing an inflammatory status via up/downregulation of costimulatory molecules differ in plaque microenvironment from the pattern of other tissues? And if yes, is there a costimulatory molecule linked or probably determining the stability or instability of plaques?** Investigation of MC/recruited immune cell interaction at a molecular level and comparing them with molecular receptors/ligands engaged in other inflammatory disorders may provide interesting results. For instance, BMMCs show an upregulation of CD1d after being exposed to alpha-galactosylceramide (αGalCer) thereafter, inducing the proliferation of iNKTs (when their CD1d molecules are loaded with αGalCer) and their release of IFN-γ, IL-13, and IL-4. However, the engagement of costimulatory molecules including CD48, CD137L, CD252 (OX40L), CD274, and CD275 hampers their ability to induce IFN-γ release from iNKTs and suppress their proliferation	[108]
** > > > What cellular and molecular mechanisms contribute to the role of MC in Kounis syndrome?** The enigma of Kounis syndrome pathophysiology and the exact link between allergy to acute coronary disorder are still unexplained. Kounis syndrome is recognized as a hypersensitivity coronary disorder and MC role has been elucidated. Three subtypes have been defined: (I) release of inflammatory mediators accompanied by coronary artery spasm, (II) release of inflammatory mediators that induce coronary artery spasm accompanied by plaque erosion or rupture, (III) coronary artery stent thrombosis, MCs, and eosinophils’ presence in the aspirated thrombus. A list of allergens is reported to have the capability of activating MCs including antibiotics or anti-inflammatory drugs, poison ivy, bee, and snake venom as well as some foods	[109, 110]
** > > > What is the role and relevance of MC-expressed MHCII in the activation of recruited T cells in plaque?** MCs are among the non-classical APCs and express MHCII molecules, which has significance in having crosstalk with T cells (MC-MHCII: TCR-T cell). It is indeed not clear how the expression of this molecule corroborates the specific immune responses. There have been mice strains lacking MHCII which can be a useful tool to determine the role of this molecule in shaping the plaque immune network. Moreover, using MC-deficient mice lacking MHCII molecules or reconstitution of MC-deficient mice from either MHCII^+/+^ or MHCII^−/−^ MCs could provide a better insight into the role of MC-expressed MHCII in mouse models of atherosclerosis	[67, 111, 112]
** > > > Can the use of MC depletors or suppressor agents be promising in controlling and stabilizing the plaques given the role of MCs in redesigning atherosclerotic plaques?** Most recently, two mAbs were used to suppress or deplete MCs. Lirentelimab (AK002) which binds to inhibitory receptor Siglec-8 on MCs and Barzolvolimab (CDX-0159) capable of binding to Ig4 and Ig5 dimerization domains of cKIT avoiding cKIT binding to its ligand SCF	[113–115]
** > > > What is the role of MMPs and TIMPs in the rupture or stability of plaques? Does profiling them as a panel in this term could be used as a biomarker to determine the stability of plaques?** MMPs are responsible for the breakdown of ECM proteins, and TIMP-1 to TIMP-4 play a role in their control. Mice express MMP-2 and MMP-9 and interestingly MCs are among the main cell types that produce MMPs and release upon degranulation. Previously, a significant boost in MMP activity in the hearts of ApoE^−/−^ mice on HFD which was accompanied by increased collagen degradation, mast cell degranulation, and death of myocytes was reported. We, therefore, suggest the reconstitution of ApoE^−/−^Kit^W−sh/W−sh^ mice with either MMP-2^+/+^ or MMP-2^−/−^ MCs (also applying the same protocol for MMP-9) to discover the effect of MC-produced MMPs on development and stability of plaques. Additionally, profiling TIMPs in humans and investigating their correlation with the stability of plaques or their level fluctuation in patients with plaque rupture along with the assessment of MMP2 and -9 levels and activity (zymography) may provide new insights into the role of these biologically significant endopeptidases	[116–119]
** > > > What are the mediators and receptors involved in the neuron/MC axis linking stress to atherosclerosis?** MCs abound around neurons and their interaction with neural system is well-studied. In recent years, stress-MC-atherosclerosis axis has been the subject of several studies. In mice, a 120 restraint stress on apoE^−/−^ mice on WTD, perivascular MC activation, circulatory levels of IL-6, and beta-hexosaminidase activity were increased and correlated with IHC findings showing activation of MCs. Additionally, in mice with established atherosclerotic lesions, a single acute stress was reported to affect plaque stability and induce intraplaque hemorrhage	[54, 86]
** > > > What are the chemokine/chemokine receptors involved in MC recruitment of other immune cells to atherosclerotic plaques in humans and mice?** MCs express a variety of chemokine receptors on one hand and release chemokines on the other hand that mediate its traffic to a certain target tissue and later mediate its complicated role in the recruitment of other cells to the site among them inflammatory cells stand out as key contributors to establishing an inflammatory status. In this regard, MCs release CXCL1 which recruits neutrophils to the site by acting through CXCR2 on neutrophils In one study, the researchers used antagonists for MC-expressed CCR3 (GW782415) which could significantly reduce the migration of MCs to studied plaques in mice. CC chemokines are among the ligands for this receptor Additionally, LTB4 acts as a chemotactic mediator for MCs; however, the recent findings showed that antagonizing its receptor expressed by MCs did not alter their numbers in plaques suggesting that LTB4 has no profound role in chemoattraction of MCs into plaque microenvironment. However, many other chemoattractants can be considered for further investigation	[20, 120–123]
** > > > Does activation of MCs via complement components locally induce their atherogenic role in the disease? If yes, what possible differences in their releasing profile do exist?** We previously reviewed the interaction between MCs and the complement system. MCs are exceptional cells in terms of the complement system as (1) they produce complement components including C3 and C5, (2) their released tryptase processes them to produce anaphylatoxins mainly C5a, and (3) MCs express receptors for complement components including C5a and become activated upon binding C5a to their expressed C5aR (CD88). Additionally, the current literature supports the role of the complement system in the pathology of atherosclerosis. Components produced by activation of the complement system were shown to predominantly increase in the intima of fibrolipidic plaques (plaques containing the signs of fibrosis including sclerosis, formation of collagen in addition to lipid accumulation); moreover an increase in components mainly C5 has been documented in the circulation	[124–126]
** > > > Do ROS activate MCs, and if so, what are the effects and connections to the prognosis of diseases with MCs in the center?** ROS can be formed by MCs when they become activated via the IgE or Dectin-1 (a pathogen recognition receptor its activation induces phagocytosis, ROS production, and production of inflammatory cytokine) pathways; however, it is unclear how other cells’ produced ROS might also activate MCs. Investigation of different types of ROS and especially the receptors and involved pathways and the association with the pathogenesis of MC-dependent diseases could be an interesting topic for further research	[127–129]
** > > > Do MC-expressed TLRs have a role in initiation and development of atherosclerosis?** There is a link between infections such as *Helicobacter pylori* and atherosclerosis. MCs express a variety of TLRs that sense the presence of pathogens. MC expression of TLR, alteration in TLR expression, and possible endo-/exogenous agonists triggering MC activation are of the aspects of poorly illuminated participation of MCs in atherosclerosis	[130–132]

## Discussion and Conclusion

Although accumulation and then further chemical modification of lipids in arteries account for the main features of atherosclerosis, the crosstalk of cells of innate and adaptive immunity and the immune responses they orchestrate contribute to the establishment and progression of atherosclerotic plaques [10]. Studying the role of tissue-resident cell types including MCs in human atherosclerosis has its difficulties. The investigation of samples from aortic plaques was largely restricted to postmortem samples obtained by autopsy. However, recently, this problem has been partially solved using carotid endarterectomy tissues to study plaques from cardiovascular patients undergoing surgery in which MCs were detected [133]. Additionally, the emergence of methods based on obtaining tissues from patients or animals, enzymatically digesting the plaques, preparation of single-cell suspension, and then studying the MCs using flow cytometry could provide further data on the role of MCs in atherosclerosis [89]. Involvement in atherosclerosis and the mechanisms through which MCs contribute to the progression and instability of the plaques are partially revealed; stimuli capable of activating these cells locally in the plaque are up to date not well-determined. Cigarette smoke and allergy are two predisposing factors of atherosclerosis, and their mechanisms of action are now partially understood. However, since cigarette smoke contains 5000–7000 chemicals [134] including nicotine [135], it is expected that more xenobiotics may possess atherogenic properties and they may engage further receptors to activate MCs. In this regard, it has been reported that cigarette smoke may activate MCs and induce histamine release which acts through endothelial-expressed H1Rs and increase the inflammation and upregulate TLR2/TLR4 and nicotinic acetylcholine receptors (NAChR) expression [136]. Besides, recent findings link allergy to atherosclerosis according to the ox-LDL potency of coactivation of both MCs and macrophages to release histamine and TNF-α respectively and contribution to the initiation and progression of plaques through endothelial activation and monocyte adhesion in atopic individuals with a high-lipid diet [137]. Investigation of the role of micro-RNAs involved in atherogenesis may elucidate new aspects of MC involvement in atherosclerosis of which miR-223 and its lower levels are associated with the progression of the plaques and worsening of the patient’s conditions [138]. miR-223 overexpression has been reported to decrease the MC production of proinflammatory cytokines mainly IL-6 [139]. Additionally, it induces autophagy in vascular SMCs which prevents their transformation to foam cells the master cells of atherosclerosis [140]. Developing ex vivo culture models may be a promising method to investigate the cytoarchitecture dynamic of plaques during different phases and study the microenvironment of the lesions with MCs therein. Two models of such ex vivo systems were developed by Lebedeva et al. and Vorobyova and colleagues by culturing circular segments of plaques on collagen rafts at the medium–air interface to supply them with oxygen that could preserve the majority of cells including fibroblasts, lymphocytes, macrophages, and SMCs [141, 142]. Human MCs release an array of proteases of which cathepsin G reacts with LDL more effectively and degrades apoB-100 (mediates the LDL uptake upon binding to LDL receptor [143]) and mediates LDL molecules fusion and increases their size from 22 to 30 nm [76]. Investigations using mouse models showed that atopic mice were more predisposed to develop atherosclerosis [4]. Promising effects of anti-allergic medication (such as MC stabilizers) in mice with atherosclerosis provided a line of evidence that MCs play a role in the pathology of the disease and our current knowledge in allergy may be useful to target MCs in the treatment of atherosclerosis [4]. Finally, we address and summarize the main features and involved molecules having a role in the MC-dependent reshaping of atherosclerotic plaques in Table 2.

**Table 2:** A summary of effects of the MC presence in atherosclerotic plaques according to the studied models

Effects of MCs on atherosclerotic plaques	Involved molecules or observed features	Studied model	Ref
**Direct effects and correlation with pathological and immunological features**			
Patients with higher intraplaque MCD were reported to have significantly more cardiovascular events during monitoring	Accumulation of MCs	Human	[27]
MCs were accumulated and degranulated mostly at immediate site of rupture when compared to adjacent atheromatous or intact areas	Accumulation and activation of MCs	Human	[22]
Higher numbers of MCs in carotid atherosclerotic lesions correlate to plaque microvessel density. MCs release several mediators that support the neovascularization within the plaques	Histamine heparin chymase tryptase	Human Mice	[27, 45, 144]
MCs act as the main source of tryptase, a protease whose expression is linked to atherosclerotic plaque hemorrhage	Tryptase	Mice	[144]
MC activation results in the inhibition of cholesterol removal from foam cells	C48/80-dependent released mediators mainly chymase	Rat	[145, 146]
MCs activate T cells in the plaque by OX40L/OX40 axis (expressed on MCs and T cells, respectively)	OX40L	Mice	[31]
MCs interact with NKT Cells (MC-CD1d/TCR-NKT) and regulate (in a protective mode) their proatherogenic function	CD1d	Mice	[10]
MCs induce the transformation of macrophages to foam cells by boosting LDL uptake	Histamine	Mice	[37]
MC-released chymase and tryptase are responsible for the induction of neoangiogenesis in atherosclerotic plaques	Chymase and tryptase	Human	[44]
MC-produced chymase and tryptase aggravate atherosclerotic lesions The numbers of tryptase^+^ and chymase^+^ MCs correlate with the percentage of collagen. Additionally, the density of tryptase^+^ MCs correlates with lipidosis	Chymase and tryptase	Human	[23]
**Indirect effects**			
Hyperlipidemia induces the expression of MHC-II on MCs, and they act as APC to activate T cells	MHC-II, CD86	Mice	[7]
MCs mediate the influx of inflammatory cells including neutrophils to plaques	CXCL1	Mice	[20]

Looking at the literature regarding the role of MCs in the pathology of atherosclerosis, one can easily find out that the basic and fundamental cellular findings regarding the presence of MCs in the pathology of atherosclerosis in terms of accumulation, activation, and protease content was acquired in the 1990s; however, the molecular involvement of MCs and their crosstalk with other immune and non-immune cells were revealed later. During the past decades using MC stabilizers and secretagogue agents, development of strategies to simulate the development of plaques in mouse models by putting them on fat-rich diets and later developing MC-deficient mice alone or ApoE knockdown mice or combined (ApoE^−/−^Kit^W−sh/W−sh^) provided an opportunity to link genetics and nutrition for having a better understanding of MC involvement in the diseases. In recent years, the emergence of new technology to study the MCs in plaques of patients such as tissue microarrays [28] and recently the introduction of monoclonal antibodies to suppress or deplete MCs brought us to the idea that it is time to rethink the role of these cells in the pathology of atherosclerosis. The Investigation the role of MCs in pathogenesis of atherosclerosis was speeded up owing to the following elements: (1) introduction of IgE-independent MC activation pathway(s); (2) highlighting the role of nerve-MC axis through results of stress/atherosclerosis in mice and human; (3) the possibility of profiling plaque-accumulated MCs using flow cytometry rather than IHC and therefore the possibility of determining the mediator and cytokine secretion profile, expressed surface markers, and crosstalk of MCs with other cells; (4) analysis of gene expression profile using single-cell RNA sequencing (scRNA); and (5) introduction of new MC suppressor or depleting agents for human model (investigated in patients with MC-driven pathologies) to study the microenvironment of plaques with or without the role of MCs. Despite all the above-mentioned achievements, we believe there are different aspects of MCs in the initiation, development, and instability of plaques in atherosclerosis addressed in Table 1 that can be considered for further studies.

## Data Availability

No data are associated with this article.

## Abbreviations

APC: Antigen presenting cell
CKD: Chronic kidney disease
CPA3: Carboxypeptidase A3
CVD: Cardiovascular disease
ECM: Extracellular matrix
GPIb: Glycoprotein Ib
GrB: Granzyme B
HFD: High fat diet
HSP-60: Heat shock protein-60
LDL: Low-density lipoproteins
LOX-1: Lectin-like oxLDL receptor-1
LPA: Lysophosphatidic acid
MC: Mast Cell
MCD: MC density
MCps: Mast cell progenitors
MI: Myocardial Infarction
mMCP-4: Mouse MC protease-4
NETs: Neutrophil extracellular traps
NF-κB: Nuclear factor-κB
NK1R: Neurokinin-1 receptor
oxLDL: Oxidized LDL
PGs: Proteoglycans
PMCs: Peritoneal mast cells
RCT: Reverse cholesterol transport
ROS: Reactive oxygen species
SCF: Stem cell factor
sIgM: Secreted IgM
scRNA: Single-cell RNA sequencing
SM: Systemic mastocytosis
SMCs: Smooth muscle cells
SP: Substance P
SRA: Scavenger receptor class A
TKIs: Tyrosine kinase inhibitors
TLRs: Toll-like receptors
VCAM-1: Vascular cell adhesion molecule-1
VWF: Von Willebrand factor
WTD: Western-type diet
